# Supplementary material for: Engineering shape memory and morphing protein hydrogels based on protein unfolding and folding
Source: Nat Commun. 2022 Jan 10;13:137. doi: 10.1038/s41467-021-27744-0 (PMC8748998; doi:10.1038/s41467-021-27744-0)
Supplement: Supplementary file 1 — Supplementary Information [file 41467_2021_27744_MOESM1_ESM.pdf]

Engineering Shape Memory and Morphing Protein Hydrogels Based on Protein Unfolding and Folding

Qingyuan Bian, Linglan Fu and Hongbin Li*
Department of Chemistry
University of British Columbia
Vancouver, BC V6T 1Z1
Canada

Supplementary Information

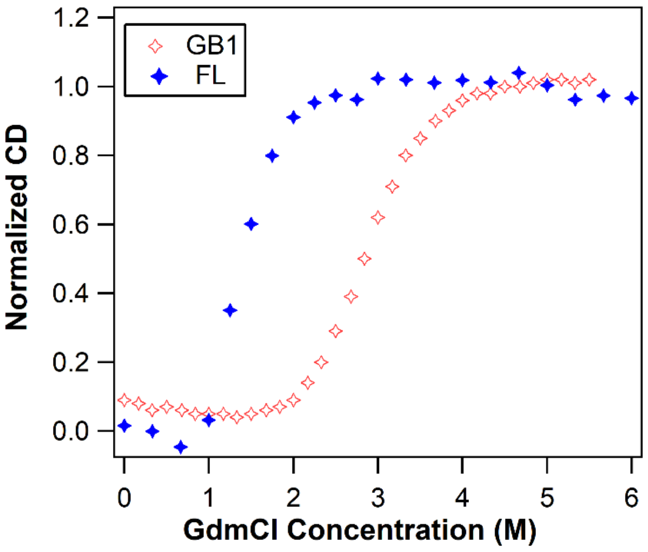

Supplementary Figure 1. Equilibrium chemical denaturation curves of FL and GB1. The GB1 data was from Ref. 34 and the data of FL was from Ref. 29.

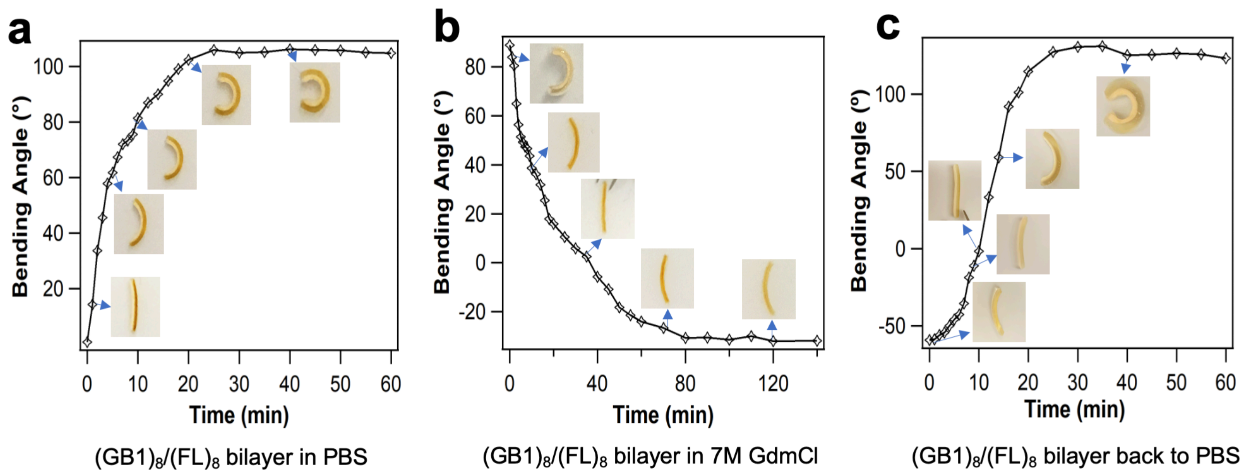

Supplementary Figure 2. Morphing kinetics of the $(GB1)_8/(FL)_8$ bilayer hydrogel strip. In **a** the hydrogel strip was directly taken out of the mold and put in PBS; in **b**, the equilibrated hydrogel strip was transferred to the buffer containing 7M GdmCl; and in c) the equilibrated hydrogel strip was put back in PBS. Between **a** and **b**, the hydrogel strip was equilibrated in PBS overnight, and between **b** and **c**, the hydrogel was equilibrated in 7 M GdmCl overnight. It is clear that there was a much slower equilibration process in both cases. However, during this slower process, the change of the bending angle was only ~15-20% of the total amplitude. The dimension of the hydrogel strip taken out of the mold was 10 mm × 3.0 mm × 1.2 mm (length × width × depth).

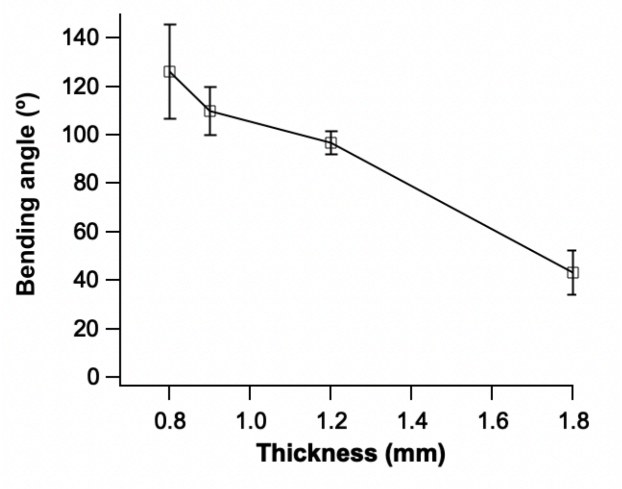

Supplementary Figure 3. Bending angle of the $(GB1)_8/(FL)_8$ bilayer hydrogel strip as a function of the total thickness of the bilayer. Error bars represent standard deviations.

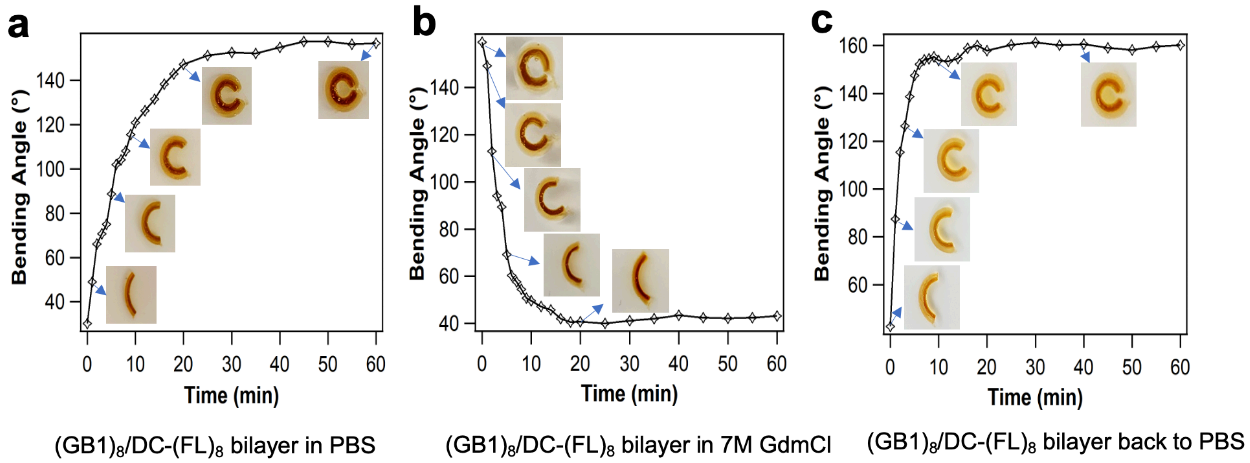

Supplementary Figure 4. Morphing kinetics of the $(GB1)_8$/DC-$(FL)_8$ bilayer hydrogel strip. In **a** the hydrogel strip was directly taken out of the mold and put in PBS; in **b** the hydrogel strip was put in 7M GdmCl; and in **c** the hydrogel strip was put back in PBS. There was no further equilibration between **a** and **b**, or **b** and **c**. The dimension of the hydrogel strip taken out of the mold was 10 mm × 3.0 mm × 1.2 mm (length × width × depth).

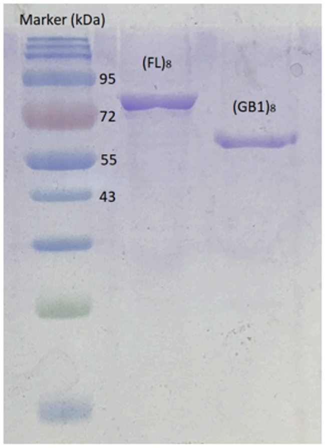

Supplementary Figure 5. 12% Coomassie blue G250 stained SDS-PAGE gel showed the identity and high purity of the obtained $(GB1)_8$ and $(FL)_8$ proteins.

$$2\text{tyrosine} + S_2O_8^{2-} \xrightarrow[\text{Light}]{[Ru(bpy)_3]^{2+}} \text{dityrosine} + 2SO_4^{2-} + 2H^+$$

Supplementary Figure 6. Scheme of $[Ru(bpy)_3]^{2+}$-mediated tyrosine coupling to form a dityrosine adduct.

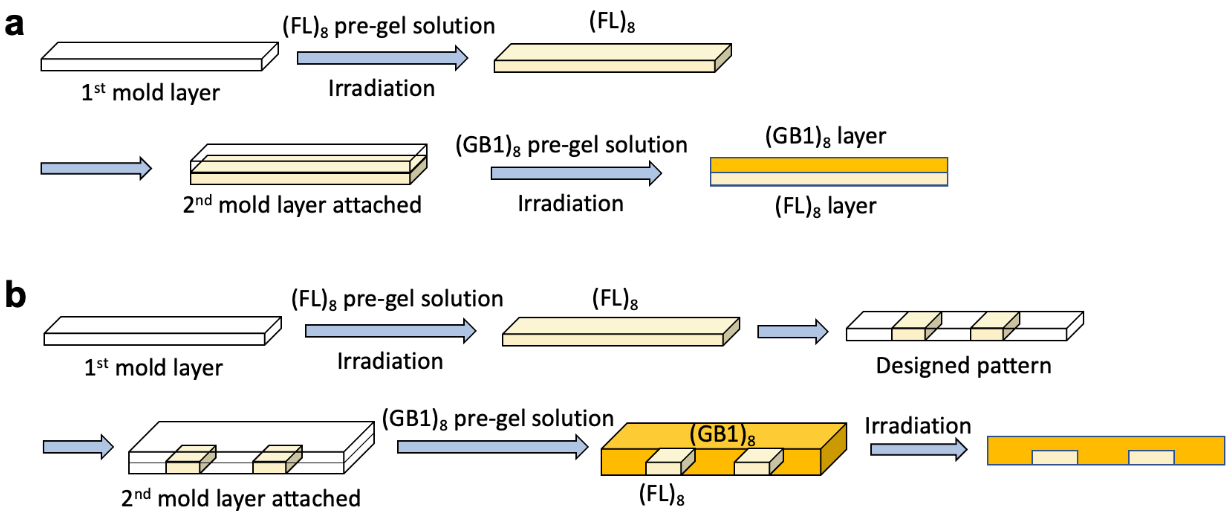

Supplementary Figure 7. **a.** Preparation of $(GB1)_8/(FL)_8$ bilayer hydrogels using a layer-by-layer gelation method. **b.** Preparation of patterned hydrogels with $(GB1)_8$/DC-$(FL)_8$ bilayer hinges. The DC-$(FL)_8$ layer was first formed and carved into a designed pattern. The $(GB1)_8$ hydrogel was then formed to achieve an integrated structure.

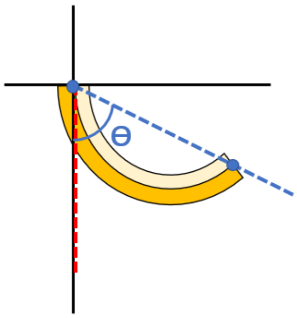

Supplementary Figure 8. The measurement method of bilayer strip bending angle, which is define as the degree of deviation (θ) from the original linear position (the red dashed line).

Supplementary Table 1. Young's moduli and swelling ratios of $(FL)_8$ hydrogels in PBS. Data is presented as average ± standard deviation.

	Young modulus (kPa) (n=3)	Swelling ratio (n=3)
10% $(FL)_8$	7.4 ± 1.2	-6 ± 3%
15% $(FL)_8$	10.5 ± 0.8	-8 ± 3%
20% $(FL)_8$	16.3 ± 1.2	-12 ± 3%
