# Supplementary material for: Engineering shape memory and morphing protein hydrogels based on protein unfolding and folding
Source: Nat Commun. 2022 Jan 10;13:137. doi: 10.1038/s41467-021-27744-0 (PMC8748998; doi:10.1038/s41467-021-27744-0)
Supplement: Supplementary file 3 — Description of Additional Supplementary Files [file 41467_2021_27744_MOESM3_ESM.pdf]

Description of Additional Supplementary Files

File Name: Supplementary Movie 1; Description: Video of the morphing of a straight protein hydrogel strip into a triangle when put in PBS (played at 60x speed).

File Name: Supplementary Movie2; Description: Video of the morphing of a triangle-shaped protein hydrogel strip into a staple shape when put in 7.0 M GdmCl (played at 60x speed).
